# Relationship Between Love Attitudes, Attachment Styles, and Personality Traits in Women Who Have Experienced Domestic Violence

**DOI:** 10.31083/AP44607

**Published:** 2025-07-08

**Authors:** Selin Balki Tekin, Ayşe Nur İnci Kenar, Hande Şenol

**Affiliations:** ^1^Clinic of Psychiatry, Denizli State Hospital, 20010 Denizli, Turkiye; ^2^Department of Psychiatry, Faculty of Medicine, Pamukkale University, 20160 Denizli, Turkiye; ^3^Department of Biostatistics, Faculty of Medicine, Pamukkale University, 20160 Denizli, Turkiye

**Keywords:** domestic violence, personality traits, attachment styles, attitudes towards love, women

## Abstract

**Objective:**

This study aimed to examine the relationship between attitudes toward love, attachment styles, and personality traits in women who have experienced domestic violence (DV).

**Methods:**

The study consisted of 64 women who experienced DV and 64 women without such history. All participants completed a sociodemographic data form and three assessment scales.

**Results:**

Among women who were exposed to violence, attitudes toward altruistic and passionate love were significantly higher (*p* < 0.001, *p* = 0.026). In contrast, women who had not experienced violence showed higher levels of friendly and possessive love attitudes (*p* = 0.010, *p* < 0.001). Women who experienced violence also exhibited a significant increase in both anxious and avoidant attachment styles (*p* < 0.001 and *p* < 0.001), while the emotionally balanced personality trait was significantly lower (*p* = 0.006). Additionally, positive correlations were found between altruistic love and anxious attachment style and between logical love and anxious attachment style (*p* = 0.021, r = 0.288; *p* = 0.033, r = 0.267, respectively). Conversely, negative correlations were observed between altruistic love attitudes and both extraversion and emotional stability (*p* = 0.038, r = –0.261; *p* = 0.030, r = –0.271, respectively), between game-playing love attitude and conscientiousness and emotional stability (*p* = 0.046, r = –0.250; *p* = 0.027, r = –0.277, respectively), and between passionate love attitude and emotional stability (*p* = 0.009, r = –0.323). Furthermore, a positive correlation was noted between friendly love attitudes and agreeableness (*p* = 0.017, r = 0.296).

**Conclusions:**

Individuals with high levels of emotional stability, along with friendly and possessive love attitudes, may be better equipped to cope with violence, while those with anxious and avoidant attachment styles, as well as altruistic and passionate love attitudes, may have difficulty handling such experiences.

## Main Points

∙ This study examined the relationship between attitudes towards love and attachment styles and personality traits in women subjected to domestic violence.

∙ Emotional stability, no comma personality traits, and friendly and possessive love attitudes may be a protective factor against violence and/or a facilitating factor in coping with violence.

∙ Anxious and avoidant attachment styles and altruistic and passionate love attitudes make it difficult to cope with domestic violence.

## 1. Introduction

Violence against women is a persistent human rights violation that exists in varying forms and is prevalent across different countries. One of the most common and yet often hidden forms of violence against women is domestic violence (DV) [1].

In the past years, studies also revealed that approximately one-third of women have experienced physical violence and 80% have been subjected to verbal abuse by their partners at some point in their lives [1, 2]. As indicated by the findings of the research project on domestic violence against women in Turkey, the prevalence of domestic physical or sexual violence against women was reported to be 38% in 2014 [3]. Factors such as low self-esteem, financial problems, excessive alcohol consumption, unemployment, mental health disorders, exposure to violence during childhood, and tolerance of violence by family members have been identified as contributing to the perpetration of violence against women [4].

John Alan Lee’s theory of love is based on an extensive review of literature and the synthesis of interviews with individuals of various ages and genders [5]. He argues that love is not an instinctive feeling, but rather a learned state. Love styles can change and be preferred. For instance, passionate love begins with physical attraction and is based on sexual intimacy [6]. On the other hand, the game-playing love style is characterized by low commitment and a focus on fun relationships [5]. The friendly love is grounded in similarities between partners, requires mutual protection, and develops gradually over time [7]. In contrast, logical love, is experienced by partners who believe they can sustain a relationship and provide a positive, fulfilling future [8]. Possessive love, is a partially pathological love style, characterized by jealousy and insecurity, whereas altruistic love is marked by individuals who love their partner despite their flaws and mistakes and prioritize their partner’s well-being [9]. To the best of our knowledge, there is no study directly examining the relationship between love attitudes and domestic violence in the English literature, but a study conducted in France in 2018 with 235 participants investigated the processes underlying the relationship between love and attitudes towards violence. This study analyzed the relationship between a person’s commitment to love and the justification of partner violence (perceived extent of violence, blaming the victim exoneration of the perpetrator, etc.). It also examined the impact of patriarchal ideologies, such as sexism and domestic violence myths, on these relationships. The results indicated that as the participants’ love increased, their tendency to blame the victim and acquit the perpetrator also increased. Additionally, a positive connection has been found between the intensity of romantic love and having patriarchal ideologies [10]. Love should be considered an important psychosocial factor in understanding the persistence of partner violence and the reasons why women who experience such violence remain in the relationship and delay seeking help.

Attachment, as defined by Bowlby, is a deep bond that individuals form with important people in their lives due to their need for trust and belonging [11]. Numerous studies have shown that attachment styles can predict behavior in relationships and potential outcomes, such as relationship satisfaction, separation, and divorce [12, 13]. In fact, insecure attachment has been found to increase the risk of becoming a victim of domestic violence. Research found that individuals with insecure attachment styles face a higher likelihood of partner violence, while those with secure attachment styles tend to have a lower risk [13].

The study of personality traits has been a topic of interest in research due to their potential to predict attachment styles and the quality of relationships [14]. The literature suggests a correlation between violence against women and certain specific personality traits. Additionally, the study also found that women who have experienced violence tend to have lower self-confidence and problem-solving abilities compared to those who have not [15].

Furthermore, research has highlighted the development of different behavioral patterns in response to violence among women, influenced by their personality traits [16]. It is widely recognized that empowering women socially, economically, and psychologically reduces their vulnerability and the risk of violence against them. Therefore, it is crucial to enhance women’s self-confidence, self-sufficiency, communication skills, coping styles, and spiritual resilience [17].

This information can then be utilized in supportive therapy sessions or violence prevention programs for these individuals. While there have been some studies exploring the link between violence and psychopathology, personality traits, and attachment styles, there is a lack of research in the English literature that examines love attitudes and all three variables together in women who have experienced domestic violence [10, 13, 15].

Therefore, this study aims to investigate the relationship between love attitudes, attachment styles, and personality traits in women who have experienced domestic violence. It is believed that understanding these characteristics can provide valuable insights into the supportive psychotherapy process for women who have experienced violence.

## 2. Methods

### 2.1 Power Analysis

It was observed that the effect size obtained in the study taken as a reference to the study was low (r = –0.305). Based on the observed effect size, a power analysis was conducted, and it was determined that at least 62 participants were needed to achieve 80% power at a 95% confidence level. Although a control group was planned, no previous studies compared a control group with a case group. Considering the expected effect size, a subsequent power analysis determined that at least 128 participants (64 in each group) were needed to achieve 80% power at a 95% confidence level.

### 2.2 Procedure

The study recruited 64 heterosexual women between the ages of 18 and 45, experiencing domestic violence, from a university hospital psychiatric clinic between April and September 2022. As a control group, 64 heterosexual women volunteers between the ages of 18 and 45 who had not experienced domestic violence, were included. All the participants were informed about the study and gave their consent. Participants with mental retardation, psychotic disorders, or bipolar disorder with a manic episode were excluded from the study. All participants underwent one-on-one clinical interviews, which included information about the definition of violence and types of violence (physical, sexual, verbal, emotional, and economic). Participants in the study group completed a sociodemographic data form that included questions about the nature of violence they had experienced, while the control group completed a sociodemographic data form without questions about violence. Participants in both groups completed three scale forms: the Attitudes Towards Love Scale, the Adult Attachment Style Scale, and the Five-Factor Personality Inventory–Short Form.

### 2.3 Measures

#### 2.3.1 Sociodemographic Data Form

This 49-item questionnaire was developed by researchers to assess a range of sociodemographic characteristics, including the participants’ and their spouses’ backgrounds, marital history, and experiences with psychiatric consultations and suicidal thoughts. Additionally, the questionnaire addressed topics related to violence, such as the types of violence experienced, reactions to violence, and sources of support sought.

#### 2.3.2 Attitudes Towards Love Scale Short Form

The Love Attitudes Scale-Short Form (LAS-SF) was developed by Hendrick, Hendrick, and Dicke (1998) based on Lee’s love dimensions [18]. Turkish validity and reliability study of the scale was conducted by Büyükşahin and Hovardaoğlu [6]. It consists of 24 items; six subscale scores, each containing four items, can be calculated from the 5-point Likert-type scale. These sub-dimensions are called gameplaying love, possessive love, passionate love, friendly love, logical love, and altruistic love. Each subscale yields a score ranging from a minimum of four and a maximum of twenty points.

#### 2.3.3 Adult Attachment Style Scale

Each attachment style is represented by six items, and the attachment style with the highest score determines the individual’s attachment style. Turkish validity and reliability study of the scale was conducted by Kesebir *et al*. [19]. The scale includes three sub-dimensions named Avoidant Attachment (1,2,5,6,15,17), Anxious Attachment (3,4,7,13,14,16), and Secure Attachment (8,9,10,11,12,18).

#### 2.3.4 Five-Factor Personality Inventory – Short Form

The Five-Factor Personality Inventory – Short Form (5FPI-SF) developed by Tatar, contains 50 items, 24 of which are reverse-scored [20]. Each item is rated on a five-point Likert scale, with 1 indicating “Not at all appropriate” and 5 indicating “Very appropriate”. The inventory comprises five sub-dimensions conscientiousness, emotional stability, extraversion, agreeableness, and intellect—with 10 items in each dimension.

### 2.4 Statistical Analysis

Data were analyzed using SPSS v25.0 (Armonk, NY, USA: IBM Corp.). Continuous variables were presented as mean ± standard deviation, median (25th and 75th percentiles). Categorical variables were reported as numbers and percentages. Assumptions for the parametric test were verified and when met, the independent samples *t*-test was used for comparisons between groups. In cases where parametric test assumptions were not satisfied, the Mann-Whitney U test was used. Chi-square analysis examined differences between categorical variables, while Spearman correlation analysis was used to explore relationships between continuous variables. A *p*-value of <0.05 was considered statistically significant.

## 3. Results

### 3.1 Sociodemographic and Clinical Characteristics

The average age of women exposed to violence was found to be 34.8 ± 6.5, while those not subjected to violence had an average age of 37.0 ± 6.0 (*p* > 0.05). In terms of education level, 23.4% of women who experienced violence had a primary school education, 39.1% had a high school education, 32.8% had a university education, and 4.7% had a postgraduate degree. Conversely, among women who did not experience violence, 9.4% had primary school education, 21.9% had high school education, 57.8% had university education, and 10.9% had postgraduate degrees. There was a significant statistical difference in education levels between the two groups (*p* = 0.005).

In terms of marital status, 50% of women who experienced violence were married, 10.9% were widowed, 35.9% were divorced, and 3.1% were living separately. In contrast, all women who did not experience violence were married. There was no significant difference in employment status between the two groups (*p* > 0.05), with 20.3% of women who experienced violence being housewives or not working, and 21.9% of women who did not experience violence being in the same category.

In terms of suicide attempts, 34.4% of women who experienced violence reported having attempted suicide, compared to only 3.1% of women who did not experience violence. This difference is statistically significant (*p* < 0.001). Similarly, a higher percentage of women who experienced violence (67.2%) had a history of psychiatric admissions, compared to 48.4% of women who did not experience violence, with this difference also being statistically significant (*p* = 0.032).

### 3.2 Analyses of Love Attitudes, Attachment Styles, and Personality Trait Scales

Regarding love styles, women who experienced violence had significantly higher scores in both altruistic love and passionate love (*p* < 0.001, *p* = 0.026, respectively), while women who did not experience violence had higher scores in friendly and possessive love (*p* = 0.010, *p* < 0.001, respectively) (See Table 1). When examining attachment styles, women who experienced violence scored significantly high in avoidant and anxious attachment (*p* < 0.001), whereas no significant differences were observed in secure attachment scores (*p* > 0.05) (Table 2). In the context of personality traits, women who did not experience violence had significantly higher scores in emotional stability (*p* = 0.006). However, no significant differences were observed for extraversion, agreeableness, conscientiousness, and intelligence (*p* > 0.05) (Table 3).

**Table 1.** **Comparison of attitudes towards love scale means of the groups**.

Attitudes Towards Love	Experienced DV		Didn’t Experience DV		*p*
	Mean ± SD	Med (IQR)	Mean ± SD	Med (IQR)	
Altruistic Love	12.53 ± 2.98	13 (11–15)	10.06 ± 2.56	10 (8.25–12)	<0.001* (z = –4.857)
Friendly Love	12.95 ± 3.05	13 (10.25–15)	14.34 ± 2.40	14 (13–16)	0.010* (z = –2.564)
Passionate Love	11.92 ± 2.89	12 (10–14)	10.78 ± 2.35	11 (9–12.75)	0.026* (z = –2.23)
Logical Love	11.31 ± 3.10	11 (9–14)	12.34 ± 2.56	12 (11–14)	0.061 (z = –1.872)
Game Playing Love	9.53 ± 2.89	9 (8–11)	9.27 ± 2.30	9 (8–11)	0.831 (z = –0.214)
Possessive Love	10.09 ± 3.84	10 (7.25–13)	12.31 ± 2.36	13 (11–14)	<0.001* (z = –3.829)

DV, domestic violence; SD, standard deviation; IQR, interquartile range; Med (IQR), Median (25th–75th percentiles); z, Mann Whitney U test. *p < 0.05 statistically significant difference.

**Table 2.** **Comparison of adult attachment styles scale means of the groups**.

Attachment Styles	Experienced DV		Didn’t Experience DV		*p*
	Mean ± SD	Med (IQR)	Mean ± SD	Med (IQR)	
Secure Attachment	2.80 ± 1.61	3 (1–4)	3.28 ± 1.57	3 (2–5)	0.112 (z = –1.588)
Avoidant Attachment	3.86 ± 1.65	4 (3–5)	2.83 ± 1.60	3 (2–4)	<0.001* (z = –3.591)
Anxious Attachment	3.02 ± 1.68	3 (2–4)	1.19 ± 1.07	1 (0–2)	<0.001* (z = –6.119)

*p < 0.05 statistically significant difference.

**Table 3.** **Comparison of five-factor personality inventory means of the groups**.

Personality subdimensions	Experienced DV		Didn’t Experience DV		*p*
	Mean ± SD	Med (IQR)	Mean ± SD	Med (IQR)	
Extraversion	30.55 ± 7.14	30 (25.25–35)	30.97 ± 6.97	32 (25.25–36)	0.736 (t = 0.338)
Agreeableness	38.64 ± 5.15	38.5 (35–42)	38.41 ± 5.95	38.5 (34–43)	0.812 (t = –0.238)
Conscientiousness	39.80 ± 5.50	40 (35–44)	40.03 ± 5.84	40 (37–44)	0.815 (t = 0.234)
Emotional Stability	27.08 ± 7.69	27 (22–32.75)	30.83 ± 7.46	31 (25.25–36)	0.006* (t = 2.799)
Intellect	36.05 ± 5.90	36 (31.25–40.75)	35.25 ± 4.41	34 (32–38)	0.338 (z = –0.958)

t, independent samples *t* test. *p < 0.05 statistically significant difference.

### 3.3 Correlation of Love Attitudes Scale With Attachment Styles and Personality Traits Scales

Among women who experienced violence, there was a positive correlation between altruistic love and logical love attitudes with anxious attachment (*p* = 0.021, r = 0.288, *p* = 0.033, r = 0.267, respectively) (Table 4).

**Table 4.** **Correlation of the attitudes towards love scale and the adult attachment styles scale in women experienced domestic violence**.

Attitudes Towards Love		Attachment Styles		
		Secure Attachment	Avoidant Attachment	Anxious Attachment
Altruistic Love	r	–0.088	0.126	0.288
	*p*	0.489	0.322	0.021*
Friendly Love	r	0.103	0.001	0.196
	*p*	0.416	0.991	0.120
Passionate Love	r	–0.133	0.057	0.242
	*p*	0.295	0.656	0.054
Logical Love	r	–0.033	–0.115	0.267
	*p*	0.797	0.364	0.033*
Game Playing Love	r	0.086	0.054	0.244
	*p*	0.500	0.672	0.052
Possessive Love	r	0.057	0.025	0.223
	*p*	0.656	0.846	0.076

*p < 0.05, Spearman correlation analysis was performed.

Additionally, a significant positive correlation was found between friendly love and agreeableness (*p* = 0.017, r = 0.296). There were also significant negative correlations between passionate love and emotional stability (*p* = 0.009, r = –0.323), as well as between altruistic love attitudes and both extraversion and emotional stability traits (*p* = 0.038, r = –0.261; *p* = 0.030, r = –0.271, respectively). Similarly, significant negative correlations were observed between game-playing love attitude and conscientiousness and emotional stability (*p* = 0.046, r = –0.250; *p* = 0.027, r = –0.277, respectively) (Table 5).

**Table 5.** **Correlation of the attitudes towards love scale and the five-factor personality inventory-short form in women experienced domestic violence**.

Attitudes Towards Love		Personality subdimensions				
		Extraversion	Agreeableness	Conscientiousness	Emotional Stability	Intellect
Altruistic Love	r	–0.261	0.068	0.023	–0.271	–0.024
	*p*	0.038*	0.592	0.858	0.030*	0.853
Friendly Love	r	–0.053	0.296	–0.034	–0.059	–0.163
	*p*	0.676	0.017*	0.793	0.645	0.198
Passionate Love	r	–0.117	–0.068	0.020	–0.323	–0.174
	*p*	0.356	0.591	0.872	0.009*	0.168
Logical Love	r	0.045	0.169	–0.036	0.002	0.011
	*p*	0.724	0.182	0.777	0.985	0.930
Game Playing Love	r	–0.151	0.162	–0.250	–0.277	0.068
	*p*	0.232	0.200	0.046*	0.027*	0.594
Possessive Love	r	–0.056	0.162	–0.199	–0.065	0.002
	*p*	0.659	0.201	0.116	0.610	0.989

*p < 0.05, Spearman correlation analysis was performed.

## 4. Discussion

To our knowledge, no study in English literature has used an integrative approach to examine the relationships between love attitudes, attachment styles, and personality traits in women who have experienced DV and those who have not. Since this study is among the first of its kind, it is particularly noteworthy.

Women who have experienced violence often have lower education levels, higher rates of suicide attempts, and increased psychiatric admissions. This is consistent with previous studies indicating that lower educational attainment is a risk factor for experiencing violence [21, 22, 23]. In a study conducted at the Center for Preventing Violence against Women in Ankara, revealed that most of the women in the sample had low educational attainment. Among those who were illiterate or had dropped out of primary school, the rate of physical or sexual violence was 43% compared to only 21% among those with a high school education or higher [5]. This suggests that lower education levels may hinder women’s ability to fight against violence or gain financial independence, making them economically dependent on their partners. Additionally, low education may lead some women to accept the violence they face due to feelings of helplessness [24]. However, according to Moore and Salkowe, education does not necessarily make a difference in the violence experienced by women; rather those with higher education tend to be more successful in leaving violent situations. Education is crucial for empowering women by enhancing their self-esteem, and confidence and discover new opportunities [25].

Similarly, literature indicates that suicidal tendencies are more common among women who have experienced intimate partner violence [21, 22, 26, 27]. One study reported that 30% of women who experienced sexual and physical violence contemplated suicide [5]. This could be linked to negative self-perception and feelings of helplessness among women who have experienced DV [28]. In our study, consistent with the existing literature, 34.4% of women reported suicide attempts while 67.2% had been admitted to psychiatric facilities. While no similar study has been found regarding women’s attitudes towards love in the context of DV, we did encounter research on intimate partner violence with findings that conflict with ours.

This study reported that women who were exposed to violence in dating relationships often exhibited possessive and game-playing love attitudes. This possessive attitude may lead to a tendency to become violent. The authors suggested that this difference may be related to the samples included in the study, as well as Lee’s idea that love attitudes can be shaped by relationship experiences and partner behavior [29]. This study found that women who have experienced violence often display a passionate and altruistic type of love, taking all kinds of risks for their relationships and believing that their love can overcome any obstacle. They may see the control and power their husbands use against them as a sign of the romantic love they feel for them, giving, forgiving, and loving the other despite all their mistakes [30]. While romantic love may be desirable for sharing warmth, security, and protection, it can also mask behaviors indicative of domestic violence. Understanding the complex nature of how women’s desires figure into the discourse of romantic love is important for preventing and reducing intimate partner violence [31].

The prevalence of friendly and logical love types among individuals who have not experienced violence may be linked to their tendency to choose partners similar to themselves – those who can contribute to a positive future, and share their values, allowing them to enjoy meaningful time together. This study concluded that women who have experienced violence tend to exhibit more anxious and avoidant attachment styles compared to those who have not, aligning with existing literature [13, 32]. Women with an avoidant attachment style often harbor negative expectations about their relationships, struggle to establish closeness and trust, and may feel overwhelmed by emotional burdens. As the level of anxious attachment increases in participants, so does their tendency to adopt helpless and submissive coping styles in response to problems [33]. Additionally, individuals with anxious attachment have a strong desire for close relationships, with their greatest fear being rejection. The fear of feeling unwanted or abandoned has been suggested as a potential risk factor for experiencing violence among women [34].

While previous studies have largely focused on the personality traits of the perpetrators, some findings indicate that women who have experienced violence often display more passive interpersonal attitudes and adhere to traditional gender roles [10, 33]. However, this study, did not find a significant relationship between personality traits and exposure to violence among women. Instead, it was noted that women who have not experienced violence generally tend to have emotionally balanced personality traits, which may serve as a protective factor. This could be attributed to their calm and logical reactions, making them less susceptible to emotional fluctuations. This highlights some significant findings: as altruistic and logical love attitudes increase in women, who have experienced violence, their levels of anxious attachment also rise. Women with an altruistic love style often accept their partners as they are and make sacrifices due to a fear of abandonment. This propensity contributes to the challenges many women face when attempting to leave abusive relationships. The relationship between rational love and anxious attachment can be explained by the fact that women who are afraid of taking risks and losing their partner often conform to societal expectations leading them to seek partners rationally. Furthermore, among women who have suffered domestic violence, an increase in altruistic love attitudes correlates with decreased levels of emotional stability and extraversion. Likewise, an increase in playful love attitude is associated with a decline in emotional stability and conscientiousness. These trends stem from the self-sacrificing and forgiving nature of altruistic love, which can result in social isolation and difficulties in coping with violence and emotional stress. Interestingly a 2019 study involving university students found no significant relationship between altruistic love and any personality type [12]. This difference can likely be attributed to the distinct samples used in the studies. The less binding and more pleasure-focused nature of playful love may have influenced those results. Moreover, in alignment with the findings from the same study conducted on university students, this research reveals that as the level of a passionate love attitude increases, emotional stability traits tend to decrease. In contrast, as friendly love increases, agreeableness traits also rise among women who have experienced violence. This pattern suggests that individuals in passionate love prioritize physical attraction and may make impulsive decisions, leading to heightened emotions and stress.

### Limitations

The sample size is small, which may limit the generalizability of the findings. The data collection forms rely on self-reporting, which may introduce bias. The participants’ responses may be subjective, which could affect the accuracy of the results. Although relatively severe psychiatric patients such as bipolar disorder, schizophrenia, and mental retardation were excluded from the study, not considering the psychiatric diagnoses of the participants at the time of admission (if any) according to DSM-V is a limitation as it may affect the results. Another limitation of our study is that scale correlations were not analyzed in the group of women who were not exposed to violence.

## 5. Conclusions

It is thought that certain personality traits and love attitudes may be protective or hindering factors in coping with violence. Mental health professionals can use these findings to support women exposed to violence and help them increase their mental self-defense, self-confidence, self-esteem, and communication skills. It is hoped that this study will contribute to the capacity of women victims of domestic violence to cope with and overcome violence.

## Data Availability

The data associated with the paper are available from the corresponding author upon reasonable request.
